# Another pandemic: over-preparedness is better

**DOI:** 10.1016/j.lansea.2023.100249

**Published:** 2023-07-14

**Authors:** The Lancet Regional Health – Southeast Asia

Globally, the COVID-19 pandemic is waning, with fewer reports of new cases. But the question arises whether the WHO South-East Asia Region (SEAR) is prepared for another deadly virus. The highly transmissible omicron variant caused less severe disease than earlier variants and there was more vaccine availability at this stage of the pandemic. But the delta variant, which caused increased deaths in the region, should not be forgotten. At the 76th World Health Assembly held in May, WHO Director-General Dr Tedros Adhanom Ghebreyesus cautioned about the looming threat of another deadly variant, and SEAR must prepare itself.

The shortage of medical staff in the region is of major concern. Many nurses migrate from India and Thailand for better opportunities abroad. Governments have introduced laws, incentives, and pay hikes to protect, retain, and motivate their workforce; however, their success remains to be seen. Most importantly, if the health workforce is not compensated well for their long working hours, heavier workloads, and risk of infection and death, further shortages of medical staff are inevitable. During the COVID-19 pandemic, availability of medical oxygen was a critical factor in preventing mortality, which emphasises the importance of an oxygen management plan. Such measures include, for example, decentralised procedures for oxygen procurement and allotment of a dedicated fund, where there is a conflict between central and provincial administrations.

In the beginning of the pandemic, unavailability of slots for vaccination and low supply of vaccines led to frustration among urban as well as rural populations in SEAR. During lockdown, migrant workers were stranded without jobs, money, and transportation to reach home in addition to being stigmatised as “virus-carriers”. In India, there was a considerable delay in the support to migrant workers by the government. But later, the launch of *Garib Kalyan Rojgar Abhiyaan* was launched, a scheme providing financial help and alternative skill development for migrant workers. In Thailand, some migrant workers were reluctant to get tested or vaccinated due to their illegal status. Migrant workers in Maldives, a large proportion of whom are from Bangladesh, faced abuse and exploitation—for example, being forced to work without pay. All governments must strive for vaccine equity as well as protection of migrant workers.

One may argue that the region is currently prepared with its huge capacity for vaccine production. A modelling study estimated that the first year of vaccinations in SEAR helped to avert 3,913,000 deaths due to COVID-19. But as the virus faces selection pressure due to increased population immunity, the probability of virus evolution increases too. Furthermore, use of diverse types of vaccines and booster doses make it difficult to conclude whether the overall protection imparted by vaccination is waning or not. There is also a possibility of the virus evolving undetected in immunocompromised individuals, as well as co-infection by different variants in one individual leading to emergence of recombinant lineages, such as XBB.

During the pandemic, governments tried to strike a balance between protection of their citizens and generation of revenue. At times, overriding the preference of the public is unavoidable. But the general public is also becoming less sensitive about intermittent rises in COVID-19 cases. 2 years of restrictions and breathing through a mask had mentally exhausted the public. It was unfair for the public to lose their freedom by following COVID-19 guidelines while some politicians did not practise what they preached. Hence, a sudden emergence of a deadly variant might not be taken seriously by the public. Government-led surveys would help in understanding the difficulties faced by public. Recent data breaches in CoWIN, India's COVID-19 vaccination tracking portal, are serious, and Indian government must aim for stricter data protection policies to gain more trust of the public.

Continued partnership with the WHO South-East Asia Regional Office is also important. The tenure of Dr Poonam Khetrapal Singh, the current Regional Director, expires in January 2024. The leadership and influence of the new Regional Director will be critical in the implementation of WHO's strategies in all countries of SEAR. Although genomic surveillance is a major challenge for several low-income and middle-income countries in SEAR, it would lead to faster detection of new SARS-CoV-2 variants of concern as well as other viruses. Like the adaptive response of the immune system becomes stronger and faster after a third dose of a vaccine, all countries must learn, adapt, and prepare for yet another pandemic.

